# Polymorphisms of CD16A and CD32 Fcγ receptors and circulating immune complexes in Ménière's disease: a case-control study

**DOI:** 10.1186/1471-2350-12-2

**Published:** 2011-01-05

**Authors:** José A Lopez-Escamez, Pablo Saenz-Lopez, Irene Gazquez, Antonia Moreno, Carlos Gonzalez-Oller, Andrés Soto-Varela, Sofía Santos, Ismael Aran, Herminio Perez-Garrigues, Águeda Ibañez, Miguel A Lopez-Nevot

**Affiliations:** 1 Otology & Neurotology Group CTS495, Department of Otolaryngology, Hospital de Poniente, El Ejido, Almería, Spain; 2 GENYO, - Centro de Genómica e Investigación Oncológica - Pfizer/Universidad de Granada/Junta de Andalucía, Granada, Spain; 3 Department of Immunology, Hospital Universitario Virgen de las Nieves, Granada, Spain; 4 Otology & Neurotology Group CTS495, Department of Biotechnology, Hospital de Poniente, El Ejido, Almeria; 5 Division of Otoneurology, Department of Otorhinolaryngology, Complejo Hospitalario Universitario, Santiago de Compostela, Spain; 6 Department of Otolaryngology, Complejo Hospitalario de Pontevedra, Spain; 7 Division Otoneurology, Department of Otorhinolaryngology, Hospital La Fe, Valencia, Spain

## Abstract

**Background:**

Autoimmune diseases with elevated circulating autoantibodies drive tissue damage and the onset of disease. The Fcγ receptors bind IgG subtypes modulating the clearance of circulating immune complexes (CIC). The inner ear damage in Ménière's disease (MD) could be mediated by an immune response driven by CIC. We examined single-nucleotide polymorphism (SNPs) in the CD16A and CD32 genes in patients with MD which may determine a Fcγ receptor with lower binding to CIC.

**Methods:**

The functional CD16A (FcγRIIIa*559A > C, rs396991) and CD32A (FcγRIIa*519A > G, rs1801274) SNPs were analyzed using PCR-based TaqMan Genotyping Assay in two cohorts of 156 mediterranean and 112 Galicia patients in a case-control study. Data were analyzed by χ^2 ^with Fisher's exact test and Cochran-Armitage trend test (CATT). CIC were measured by ELISA for C1q-binding CIC.

**Results:**

Elevated CIC were found in 7% of patients with MD during the intercrisis period. No differences were found in the allelic frequency for rs396991 or rs1801274 in controls subjects when they were compared with patients with MD from the same geographic area. However, the frequency of AA and AC genotypes of CD16A (rs396991) differed among mediterranean and Galicia controls (Fisher's test, corrected p = 6.9 × 10^-4 ^for AA; corrected p = 0.02 for AC). Although genotype AC of the CD16A receptor was significantly more frequent in mediterranean controls than in patients, [Fisher's test corrected p = 0.02; OR = 0.63 (0.44-0.91)], a genetic additive effect for the allele C was not observed (CATT, p = 0.23). Moreover, no differences were found in genotype frequencies for rs396991 between patients with MD and controls from Galicia (CATT, p = 0.14). The allelic frequency of CD32 (rs1801274) was not different between patients and controls either in mediterranean (p = 0.51) or Galicia population (p = 0.11).

**Conclusions:**

Elevated CIC are not found in most of patients with MD. Functional polymorphisms of CD16A and CD32 genes are not associated with onset of MD.

## Background

Ménière's disease (MD) is a chronic disease defined by recurrent spells of vertigo associated with sensorineural hearing loss and tinnitus or aural fullness. Different autoimmune diseases share susceptibility loci, but consistent associations with multiple autoimmune disorders have been restricted to three genes: the human leukocyte antigen (HLA) DRB1 gene, the PTPN22 gene encoding lymphoid tyrosine phosphatase LYP and the gene encoding cytotoxic T lymphocyte-associated 4 (CTLA-4) receptor [1]. Autoimmune mechanisms appear to be associated with the pathogenesis of some types of sensorineural hearing loss (SNHL), [2,3] including rapidly progressive bilateral SNHL (autoimmune inner ear disease),[4] sudden SNHL [5] and MD [6-8]. Allelic variants of the HLA class II gene DRB1 and the functional polymorphism 1858C > T of the PTPN22 gene have been associated to bilateral MD in mediterranean population, suggesting an autoimmune process [9].

Diversity of populations may explain differences in HLA-DRB1 associations found in British [10], German [11], Japanese [12], Korean [13] or Spanish patients with MD [14]. Moreover, the response to steroids therapy and the finding of elevated levels of circulating immune complexes (CIC) in some patients with MD, especially in the active phase, has supported the hypothesis of autoimmunity in MD [15,16]. A decrease in CIC clearance could determine an increase of CIC levels which are deposited in the blood vessels of the endolymphatic sac, resulting in inflammation with increase in vascular permeability and the development of endolymphatic hydrops [16].

The Fcγ receptors CD16A and CD32A connect the innate and the adaptative immune response by transmitting activating signals to natural killer lymphocytes and myeloid cell upon recognition of Fc of IgG [17].

CD32A (FcγRIIa) exhibits low affinity for monomeric IgG, but binds IgG CIC efficiently. Two genes and two transcripts of FcγRIII have been described (FcγRIIIa, and IIIb), which also bind IgG CIC and FcγRIIIa (CD16A) has intermediate affinity for monomeric IgG and it is involved in the removal of CIC [18]. CD32A is expressed in all myeloid cells, platelets, and endothelial cells, whereas CD16A is present on monocytes, macrophages, NK cells and γ/δ T cells [17].

Fcγ receptors subclasses display functionally relevant genetically determined polymorphisms. So, FcγRIIa displays a G to A single nucleotide polymorphism (SNP) at nucleotide 519 in the region specifying its ligand binding domain, causing an arginine (R) to histidine (H) amino acid substitution at position 131 (rs1801274). The FcγRIIa-H131 allotype shows higher binding efficiency for human IgG2 and IgG3 isoforms, compared to FcγRIIa-R131. The FcγRIIIa gene displays a C to A substitution in exon 4 at nucleotide 559, resulting in a valine (V) to phenylalanine (F) substitution at amino acid position 158 (rs396991) [19]. IgG-induced NK cell activity is increased among FcγRIIIa-V/V158 donors, compared to FcγRIIIa-F/F158 individuals, due to a higher affinity of the former allotype for IgG1, IgG3 and IgG4 [18,20]. These low binding phenotypes has been associated with susceptibility to recurrent viral infections, rheumatoid arthritis [21,22] and systemic lupus erythematosus [23] and are clinically relevant because they modify the clinical course and the response to therapy of several human diseases [24].

The aim of this study was to analyze the FcγRIIa*519A > G and FcγRIIIa*559A > C SNPs in the Fc receptor cluster in patients with definite MD.

## Methods

### Subjects

DNA Samples from 268 unrelated patients with MD and 770 controls from two ethnically defined regions were included in a prospective multicenter study between January 2004 and December 2009. All patients were diagnosed according to the diagnostic scale for MD of the American Academy of Otolaryngology- Head and Neck Surgery (AAO-HNS) [25]. Group 1 consisted of 156 Caucasian patients from the east and southeast areas of Spain (85 unilateral, 71 bilateral MD) that were compared with 626 controls from the same area. Group 2 was recruited to replicate the results of group 1 and included 112 (47 unilateral, 65 bilateral MD) Caucasian patients and 144 controls from Galicia (northwest of Spain). Five centers recruited samples from patients with MD for this study: Hospital La Fe from Valencia, Hospital Virgen de las Nieves from Granada, Hospital de Poniente from El Ejido, Almeria (group 1) and Hospital Santiago de Compostela and Hospital de Pontevedra (group 2). Controls for group 1 were unrelated Caucasian volunteer blood donors at Almeria and Granada; controls for group 2 were provided by the Spanish DNA BioBank http://www.bancoadn.org. The selection of controls was matched by age and gender in both populations. Caucasian subjects with a different origin (Maghreb, Romani minority) were excluded to avoid a substructure bias in the controls (Table 1).

**Table 1:** Baseline characteristics of case and control subjects

Variable	Southeast population		Northwest population	
	MD	Control	MD	Control
Subjects	156	626	112	144
Female gender (%)	53.8	59.7	50.0	51.4
Age (years)				
Mean	56.1	38.0	55.5	37.2
Median	56	37	58	38,5
Range	15-88	19-68	12-79	18-64
SD	13.1	9.5	13.8	13.1

The subject's informed consent was obtained to participate in the study according to the Declaration of Helsinki and the local Institutional Board approved the study. The clinical features and HLA-DRB1 and DQB1 genotyping of some patients with MD from both groups were previously reported [14].

### DNA extraction and genotyping

All genomic procedures were carried out at Department of Immunology, Hospital Virgen de las Nieves, Granada, Spain. Total genomic DNA was automatically isolated from peripheral blood of patients and healthy controls using the M-48 robot (Genovision) and the MagAttract DNA Blood Mini M48 (192) kit from Qiagen. The genotyping of the FcγRIIa*519A > G (rs1801274) and FcγRIIIa*559A > C (rs396991) SNPs were performed using a predeveloped TaqMan SNP Genotyping Assays (Applied Biosystems, Foster City, CA). The PCR reaction was carried out using the Assays-on-Demand SNP genotyping kit (AB). SNPs amplification assays were used according to the manufacturer's instructions. In short, 5 μl of reaction solution containing 10ng of DNA was mixed with 2.5 μl of 2× TaqMan Universal PCR Mix (AB), 0.05 μl of predeveloped assay reagent from the SNP genotyping product (AB) containing two primers and two MGB-Taqman probes and 1,45 μl of desionizated water. Reaction conditions consisted of preincubation at 50°C for 2 minutes and at 95°C for 10 minutes, followed by 40 cycles at 95°C for 15 seconds and at 60°C for 1 minute. Amplifications and analysis were performed in an ABI Prism 7500 Sequence Detection System (AB) using the SDS 1.4 software for allelic discrimination (AB).

### Determination of circulating immunocomplexes

Serum samples from 331 patients (85 mediterranean and 203 Galicia patients) were obtained to determine CIC concentrations. So, an Autostat™II test for C1q CIC kit (HYCOR, Agilent Technologies, Amstelveen, The Netherlands) was used to detect immune complexes containing both C1q and IgG in a Hytec 288 Plus System (HYCOR). Positive and negative controls and reference values were provided by the manufacturer (0-40 μg/ml).

### Statistical analysis

Allelic and genotype frequencies were calculated by direct counting. Hardy-Weinberg equilibrium was tested for each SNP among controls. For 2 × 2 contingency tables of allele frequencies of patients with MD and controls, odds ratios (ORs) and 95% confidence intervals (95% CI), χ^2 ^test and Fisher's exact test were calculated to determine the exact two-sided p-values using SPSS Software (SPSS Inc., Chicago, IL, USA). Genotype frequencies were compared for each of the 2 × 3 contingency tables by Cochran-Armitage trend test, which may have more power than Fisher's exact test if a trend exists across genotype categories under the additive genetic effect model. Probability values (*p*) were corrected by multiplying the *p *value by the number of genotypes compared. p-values lower than 0.05 were considered statistically significant.

## Results

The CD32 (FcγRIIa*519A > G) and CD16A (FcγRIIIa*559A > C) SNPs in the Fcγ receptors were selected to investigate their association with MD. Genotype distributions of both SNPs in mediterranean and Galicia controls exhibited Hardy- Weinberg equilibrium (data not shown), and the minor allele frequencies of all SNPs were over 5% in controls from both groups.

First, the frequencies of genotypes AA and AC for FcγRIIIa*559A > C were significantly different in controls from southeast and northwest of Spain (Table 2). So, the genotype AA of FcγRIIIa (low binding phenotype) is more common in Galicia than in mediterranean population (Fisher's exact test, p = 6.9 × 10^-4^). Second, we compared the genotypes distribution of both SNPs between patients with uni and bilateral MD (Table 3). There was no difference in the genotype of both FcγRIIa*519A > G and FcγRIIIa*559A > C SNPs between patients with one or both ears affected either in mediterranean or Galicia population (all, p > 0.05).

**Table 2:** Distribution of rs396991 (CD16A) and rs1801274 (CD32) genotypes and phenotypes in controls subjects from southeast and northwest populations.

Genotype	Phenotype	Southeast	Northwest	Fisher exact p values	Corrected p
CD16A		N = 626 (%)	N = 141 (%)		
AA	176F/F	221 (35.3)	73 (51.8)	2.3 × 10^-4^	*6.9 × 10*^*-4*^
AC	176F/V	314 (50.2)	54 (38.3)	7 × 10^-3^	*0.02*
CC	176V/V	91 (14.5)	14 (9.9)	0.09	NS
					
CD32		N = 349 (%)	N = 144 (%)		
AA	131R/R	95 (27.2)	28 (19.4)	0.04	NS
AG	131R/H	162 (46.4)	83 (57.6)	0.01	0.03
GG	131H/H	92 (26.4)	33 (22.9)	0.25	NS

Low affinity binding phenotypes are 176F/F and 131 R/R.

**Table 3:** Distribution of CD16a and CD32 genotypes in patients with uni and bilateral MD (χ2 test).

Southeast group	Unilateral	Bilateral	P value
CD16A genotype	N = 85 (%)	N = 71 (%)	
AA	37 (43.5)	33 (46.5)	0.91
AC	33 (38.8)	27 (38.0)	
CC	15 (17.6)	11 (15.5)	
			
CD32 genotype	N = 85(%)	N = 71(%)	
AA	21 (24.7)	22 (31.0)	0.59
AG	42 (49.4)	29 (40.8)	
GG	22 (25.9)	20 (28.2)	

			

**Northwest group**	**Unilateral**	**Bilateral**	**P value**

CD16A genotype	N = 45 (%)	N = 65 (%)	
AA	17 (37.8)	29 (44.6)	0.34
AC	22 (48.9)	28 (43.1)	
CC	6 (13.3)	8 (12.7)	
			
CD32 genotype	N = 47 (%)	N = 65 (%)	
AA	12 (25.5)	18 (27.7)	0.84
AG	25 (53.2)	36 (55.4)	
GG	10 (21.3)	11 (16.9)	

The CD32 allelic frequencies (rs1801274) did not differ between patients and controls either in southeast (p = 0.51) or northwest groups (p = 0.11). Moreover, AA and AG genotypes did not increase the risk for MD in any of both populations (Table 4).

**Table 4:** Frequency of CD32 alleles and genotypes among MD patients and healthy controls (Fisher's exact test).

Southeast	MD N = 155 (%)	Controls N = 349 (%)	OR (95% CI)	P value
AA	43 (27.7)	95 (27.2)	1,03 (0.67-1.57)	0.49
AG	70 (45.2)	162 (46.4)	0,95 (0.65-1.39)	0.43
GG	42 (27.1)	92 (26.4)	1,04 (0.68-1.59)	0.47
				
Allele	2n = 310 (%)	2n = 698 (%)	1,00 (0.77-1.31)	0.51
A	156 (50.3)	352 (50.4)		
G	154 (49.7)	346 (49.6)		

				

**Northwest**	**MD N = 112 (%)**	**Controls N = 144 (%)**	**OR (95% CI)**	P value

AA	30 (26.8)	28 (19.4)	1,52 (0.84-2.72)	0,11
AG	61 (54.5)	83 (57.6)	0.88 (0.53-1.45)	0.35
GG	21 (18.8)	33 (22.9)	0.77 (0.42-1.43)	0.26
				
Allele	2n = 224 (%)	2n = 228 (%)	1.25 (0.88-1.79)	0.11
A	121 (54.0)	139 (48.3)		
G	103 (46.0)	149 (51.7)		

In addition, no differences were found in the allelic frequency of CD16A (rs396991) in controls subjects when they were compared with patients with MD from the same geographic area. Although the genotype AC of CD16A receptor was significantly more frequent in mediterranean controls than in MD patients (OR = 0.63 (0.44-0.91), Fisher's test, corrected p = 0.02), a genetic additive effect for the allele C was not observed (Cochcran-Armitage trend test, p = 0.23). The genotype AA of FcγRIIIa (rs396991) encoding the FcγRIIIa-F/F158 phenotype was not associated with MD (95% CI, 1.04-2.10; corrected p = 0.06) (Table 5). Moreover, we replicated our observations in the northwest group, and no differences were found in genotype frequencies for rs396991 between patients with MD and controls from Galicia (CATT, p = 0.11).

**Table 5:** Frequency of CD16A alleles and genotypes among MD patients and healthy controls.

Southeast	MD N = 156 (%)	Controls N = 626 (%)	OR (95% CI)	Fisher's exact p values	Corrected p
AA	70 (44.9)	221 (35.3)	1.49 (1.05-2.12)	0.018	0.054
AC	60 (38.5)	314 (50.2)	0.62 (0.43-0.89)	0.006	0.018
CC	26 (16.7)	91 (14.5)	1.17 (0.73-1.89)	0.29	NS
					
Allele	2n = 312 (%)	2n = 1252 (%)	1.17 (0.91-1.52)	0.13	NS
A	200 (64.1)	756 (60.4)			
C	112 (35.9)	496 (39.6)			

					

**Northwest**	**MD N = 110 (%)**	**Controls N = 141 (%)**	**OR (95% CI)**	**Fisher's exact p values**	**Corrected p**

AA	46 (41.8)	73 (51.8)	0.67 (0.40-1.11)	0.07	NS
AC	50 (45.5)	54 (38.3)	1.34 (0.80-2.23)	0.15	NS
CC	14 (12.7)	14 (9.9)	1.32 (0.60-2.91)	0.31	NS
					
Allele	2n = 220 (%)	2n = 282 (%)	0.75 (0.51-1.09)	0.08	NS
A	142 (64.5)	200 (70.9)			
C	78 (35.5)	82 (29.1)			

No additive effect was observed in homozygous subjects with Cochran-Armitage trend test in the southeast (p = 0.23) and the northwest group (p = 0.14).

Circulating immunocomplexes were investigated in 331 patients with MD. There was an increase of CIC in 25 patients (7.6%). No difference was found in the frequency of elevated CIC between patients with unilateral (7%) or bilateral involvement (8.4%). In addition, we did not found association between elevated CIC and the homozygous genotype AA of FcγRIIIa either in mediterranean (p = 0.42) or Galicia (p = 0.64) patients in our series.

## Discussion

Our shows that heterogeneity within caucasian population should be consider in genetic association studies. The distribution of frequencies of CD16A genotypes AA and AC was different in Galicia and mediterranean controls. The genotype frequencies in our Mediterranean controls are identical to frequencies reported in a previous study from the same geographical area [21]. The difference of CD16A with the Galicia population may indicate a differential immunogenetic background between populations.

These two SNPs were chosen because they determine a low binding phenotype for Fcγ receptors and their consistent association with rheumatoid arthritis [21,22] and systemic lupus erythematosus [23].

The genotype AC of the FcγRIIIa*559A > C (CD16A) gene is overrepresented in mediterranean controls and it may confer some protection for MD in mediterranean patients, although the trend test did not confirm an additive effect in homozygous subjects. In addition, the genotype AA of CD16A was marginally associated with MD in mediterranean population. However, these finding could not be replicated in the population from Galicia within the same country. The allele A encodes for a V to F substitution at amino acid position 158, resulting in an FcγRIIIa allotype with a lower affinity. The lower affinity of this Fc receptor for IgG subclasses may decrease the binding efficiency for IgG1, IgG3 and IgG4 complexes, resulting in a lower clearance of CIC.

We cannot support the hypothesis that FcγR polymorphisms determine a CIC-mediated damage in the inner ear of patients with MD, although the efficiency of FcγR-IgG interaction may predict the efficacy of CIC clearance [17]. A less efficient CIC clearance may lower the threshold for complexes deposition in the vascular endothelium, with the induction of inflammation observed in autoimmune diseases as rheumatoid arthritis [20].

The rs 396991 of CD16A has been associated with rheumatoid arthritis [21,22], systemic lupus erythematosus [23,24], vasculitis [26], demyelinating disease [27], immune thrombocytopenic purpura [28] and myasthenia gravis [29]. This is the first report describing an association between MD and polymorphisms of CD16A receptor in mediterranean population, although we could not replicate this effect in the Galicia population. We also previously found that the HLA class II allele DRB1*1101 was associated in mediterranean patients with MD, but not association was found in patients from Galicia [14].

MD is multifactorial and the interaction of several genes with the environment is probably involved. Several mutations have been described in the coagulation factor C homology (COCH) gene (14q12-13), which cause the autosomal dominant SNHL DFNA9 (OMIM603196) [30], and a high prevalence of symptoms of MD has been described in European families with a P51S mutation in exon 4 of COCH gene [31]. However, these mutations were not found in patients with MD [32].

Many tests have been developed for the detection of CIC, including polyethylene glycol (PEG) precipitation and radial immunodiffusion and cellular based assays. No single procedure appears to detect all types of CIC, however, those procedures which detect CIC containing fragments of complement (e.g. C1q and C3d) appear to detect clinically relevant events. Although several reports have found elevated CIC in 54-96% of patients with MD using the PEG precipitation assay [15,16], another study using our method, a solid phase immunosorbent assay (ELISA) for C1q and IgG also found an elevation of CICs in 4% of 49 patients with MD [34]. Our results confirm this study and found elevated CIC in 7.4% of 331 patients. Moreover, subanalysis of patients with bilateral MD did not show a higher frequency of elevated CIC. Our data cannot support that pathophysiology of MD is different in patients with uni or bilateral involvement.

Histopathology studies in temporal bones from patients with MD have shown endolymphatic hydrops and vestibular fibrosis in the inner ear. Immunohistochemistry have found deposits of C3 and C1q in the membranous labyrinth located at the basal membrane, subepithelial connective tissue, vestibular ganglion and endolymphatic sac of patients with MD [35,36]. It appears that immune injury may be induced in the inner ear at sites where CIC are deposited in the vessels. It is hypothesized that deposits of CIC in the vessels of the stria vascularis may interfere with the recycling of K^+ ^ions, resulting in an osmotic imbalance, cellular stress and probably dysfunction of fibrocytes of the spiral ligament. The flux of K^+ ^ions into the endolymphatic compartment from the stria vascularis reflects the current flow underlying the endolymphatic potential [37]. In a guinea pig model, it appears that type I fibrocytes and other cells are damaged before the onset of hydrops, but the mechanism is unknown [38].

One of the limitations of this study is that we could not demonstrate an association between patients with the genotype AA of CD16A and elevated CIC. This could be explained because blood samples were not obtained in an acute phase of the disease, and CIC levels may fluctuate within the same patient according to the disease activity. Our next study will look at the CIC level during the acute phases of MD to compare them with basal CIC levels in subjects with genotype AA of CD16A.

## Conclusions

Elevated CIC were found in 7% of patients with MD. Polymorphisms of CD16A (FcγRIIIa*559A > C, rs396991) and CD32A (FcγRIIa*519A > G, rs1801274) genes are not associated with onset of MD.

## Competing interests

The authors declare that they have no competing interests.

## Authors' contributions

JALE and MLN conceived and coordinated the study and draft the manuscript. JALE, ASV, SS, IA and HPG participated in the clinical design of the study, recruited patients with Meniere's disease and collected blood samples for DNA extraction. PSL, IG and AM recruited controls and carried out the genotyping studies. CGO and AI carried out the immunoassays for immunocomplexes. JALE and IG performed the statistical analysis. All authors read and approved the final manuscript.

## Pre-publication history

The pre-publication history for this paper can be accessed here:

http://www.biomedcentral.com/1471-2350/12/2/prepub
